# An Unsupervised Approach to Structuring and Analyzing Repetitive Semantic Structures in Free Text of Electronic Medical Records

**DOI:** 10.3390/jpm12010025

**Published:** 2022-01-01

**Authors:** Varvara Koshman, Anastasia Funkner, Sergey Kovalchuk

**Affiliations:** 1 Federal Almazov North-West Medical Research Centre, Center for Artificial Intelligence Development in Medicine, 197341 Saint Petersburg, Russia; koshman.varia@gmail.com (V.K.); funkner.anastasia@gmail.com (A.F.); 2 National Center for Cognitive Research, ITMO University, 49 Kronverksky Pr., 197101 Saint Petersburg, Russia

**Keywords:** syntactical parsing, natural language processing, electronic health records, Node2Vec, automatic text labeling, graph algorithms

## Abstract

Electronic medical records (EMRs) include many valuable data about patients, which is, however, unstructured. Therefore, there is a lack of both labeled medical text data in Russian and tools for automatic annotation. As a result, today, it is hardly feasible for researchers to utilize text data of EMRs in training machine learning models in the biomedical domain. We present an unsupervised approach to medical data annotation. Syntactic trees are produced from initial sentences using morphological and syntactical analyses. In retrieved trees, similar subtrees are grouped using Node2Vec and Word2Vec and labeled using domain vocabularies and Wikidata categories. The usage of Wikidata categories increased the fraction of labeled sentences 5.5 times compared to labeling with domain vocabularies only. We show on a validation dataset that the proposed labeling method generates meaningful labels correctly for 92.7% of groups. Annotation with domain vocabularies and Wikidata categories covered more than 82% of sentences of the corpus, extended with timestamp and event labels 97% of sentences got covered. The obtained method can be used to label EMRs in Russian automatically. Additionally, the proposed methodology can be applied to other languages, which lack resources for automatic labeling and domain vocabulary.

## 1. Introduction

It has been previously shown that the performance of language machine learning models significantly increases when textual content of EMRs is included in the model’s training data [1]. However, at this point, it is barely possible to use it when working with the Russian language due to the lack of labeled datasets available. The main reason is that manual labeling requires significant effort and time by domain experts. On the other hand, an automatic annotation system can save experts’ time and promptly provide researchers with labeled data. Unfortunately, though, the idea of automatic annotation faces significant challenges for many languages, such as a lack of ready-to-use medical terminologies (e.g., terminologies of signs and symptoms, diseases, diagnosis, medications, vocabularies of medical abbreviations, etc.). Additionally, a specific syntactic structure with free word order missing conjunctions and omitting subject naming complicates the process of automatic annotation.

For clinical text processing in English, one may find extensive medical resources such as structured medical vocabularies (e.g., Unified Medical Language System (UMLS) [2], SNOMED CT), systems for clinical information extraction (e.g., cTAKES [3]), or search engines (e.g., PubMed, MetaMap [4]) are available. However, any other language except English has fewer resources to integrate into the research process. Thus, scientists search for various ways to reduce this limitation. In a project with a similar goal to automate the process of clinical text annotation in the Spanish language [5] an analogous tool to MetaMap was implemented from scratch. The tool performed the mapping of medical terms in EMRs to concepts in UMLS Metathesaurus and utilized Spanish-specific biomedical resources such as vocabularies of health acronyms and abbreviations. Researchers working with EMRs in Russian implemented a similar instrument to MetaMap on their own using MeSH, the only available vocabulary from UMLS for the Russian language [6]. However, the focus of the study was disease linking, and automatic annotation was not performed.

In case the language lacks analogous medical resources, one may use the Wikipedia-based approach to annotation which is being researched. For example, a recent study successfully applied DBpedia to link words in Arabic to their English translations in textual data [7]. To this end, researchers used morphological parsing and DBpedia’s multilingual word mapping. Wikipedia was also successfully applied in a bilingual entity linking for both Chinese and English language systems, which showed state-of-the-art performance on the task [8]. The application of a Wiki-based approach to Russian was studied by Sysoev A., who used a Russian Wikidata graph for training word embeddings to improve the performance of entity linking [9]. There are numerous studies outside the biomedical domain on this approach to annotation, for which ideas can be applied in a clinical context. For instance, J. Raiman assigned categories to words in a text using Wikidata graph’s parental relations in an entity disambiguation task [10]. This approach of labeling with Wikidata’s categories has not been applied yet for the annotation of EMRs. An attempt to extract deterministic characteristics from EMRs in Russian was proposed by A. Funkner [11]. However, results contained incorrect and unnecessary constructions, so it was concluded that syntactic and morphological parsing should be used to discover sentence structure.

A recent study on Chinese EMRs suggested an unsupervised approach linking symptoms to the ICD10 classification [12]. Faced with the same issue of a lack of structured data in a corresponding language, authors pre-compiled a vocabulary of signs and symptoms crawling data from Chinese medical websites (more than 12 k terms in size). Additionally, they utilized word embeddings pre-trained with Word2Vec to compare a mention with a term in a vocabulary in terms of semantic similarity. However, most often, embeddings are trained separately to convey the context of the study. When working with graphical structures, a Node2Vec [13] method is commonly used in fields including biomedical [14,15]. Its random sampling strategy helps to preserve hierarchical relations between nodes in word embeddings. Node2Vec method for training word embeddings was also applied to syntactic trees for text generation [16]. However, it has not been used yet for text clustering, which is the focus of the current study. Tree similarity-based text clustering was suggested for relation extraction beyond the clinical domain [17]. Using cosine similarity was rejected mainly because the relationships between words might differ in different contexts. This way, a similarity function was proposed, and the retrieved clusters were then labeled with the most frequent head of a tree. However, with Node2Vec, the mentioned limit can be overcome by incorporating syntactic relations between words in word embeddings.

This paper aims to design and develop a method for automatic detection of repetitive semantic constructions in unstructured text data of EMRs. First, we utilize morphological and syntactic parsing to get structural representations of sentences. Then we train word embeddings using the Node2Vec method and group words with similar embeddings together; we find groups of similar syntactic trees and label them with Wiki-data categories.

To the best of our knowledge, we are the first to apply Node2Vec on syntactic trees in the task of text clustering. An approach of labeling with Wikidata concepts using categorical relations is firstly applied for labeling medical text data. Additionally, this is the first tool for automatic annotation of EMRs in Russian. A significant advantage of the proposed approach is that it is universal and can easily be adapted to another language regardless of the variety of biomedical resources available for this language.

## 2. Methods

The variety of mentioned drugs, signs, and symptoms terms is not usually covered by vocabularies, as it is hard to make them complete. Additionally, EMRs usually include specific language features (e.g., word abbreviations, typos), which are hard to correct with no additional medical vocabulary of acronyms. Therefore, we group similar semantic constructions to put similar symptom terms, word abbreviations, and drug names. With this done, when we perform automatic labeling, some of the words not presented in the knowledge base get relevant labels as members of a labeled group.

**Figure 1:**
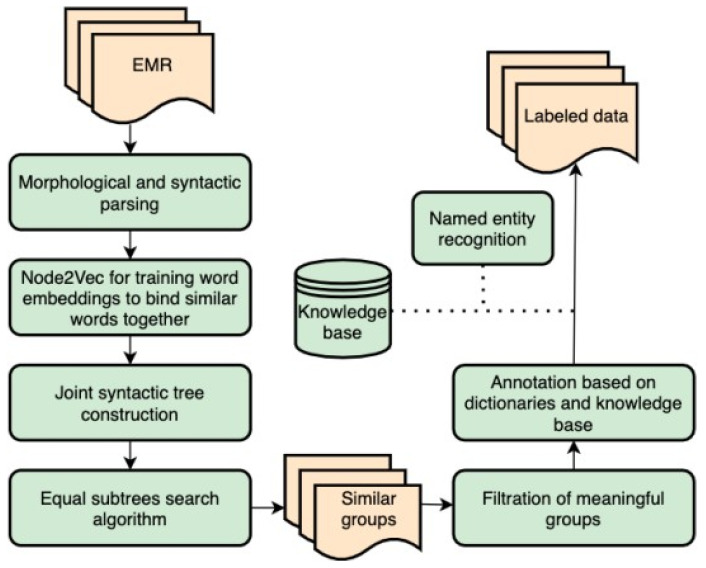
Method schema of sequential modules with EMR as input and labeled groups as output.

The detailed method schema is depicted in Figure 1. First, we split textual data of EMRs into sentences and have made morphological and syntactic analyses. With these means, we get a hierarchical structure for each sentence. Then, we applied Word2Vec [18] and Node2Vec [13] methods to train word embeddings on a corpus of syntactic trees. We have picked the most similar ones for each word and added them as new nodes on the same level to the initial tree. Cosine distance was used as a similarity metric. After these modifications to initial parsed trees, they were joined together to form one merged tree. We suggested an algorithm for finding equal subtrees, resulting in groups with similar semantic constructions. Eventually, a labeling module uses the medical knowledge base to assign labels to groups. The knowledge base is a Wikidata-based language-specific base set once before labeling.

### 2.1. Morphological and Syntactic Parsing

Syntactic and morphological analyzers are used to extract information about sentence structure. This procedure ensures that groups with similar semantic constructions share the same structural meaning. In this research, we use a neural model for morphological tagging, as it showed promising results before [19]. We utilized a high performant graph-based parser with neural attention suggested by [20] for syntactic parsing. Both approaches were implemented for the Russian language by the DeepPavlov project [21]. Several studies proved that parsing of medical text is better with a model that is also trained on medical data [22,23]. However, we did not have labeled data for re-training, so we used a model already trained on a UD Russian SynTagRus corpus (version 2.3).

**Figure 2:**
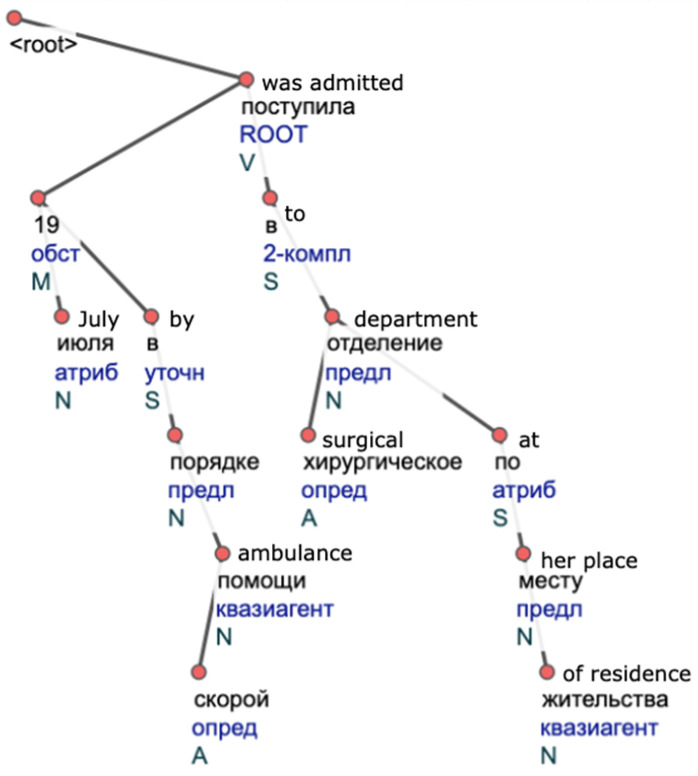
An example of a syntactic tree of a sentence “On 19 July, she was admitted by ambulance to the surgical department at her place of residence” in a CoNLL-U format.

We use pos-tags provided by a morphological analyzer to ensure similar words have the same part of speech. An example of a resulting syntactic tree in a CoNLL-U format used further in analyses has a structure shown in Figure 2. Word’s initial form, lemma, and a pos-tag are stored in a tree’s node. Syntactic relations connect semantically dependent nodes.

### 2.2. Node2Vec on Syntactic Trees

The received syntactic trees commonly contained phrases with similar meanings yet said in different words. We aimed to cluster these similar fragments of trees and apply labeling to groups instead of single ones to let the annotation cover more text. To compare words in terms of similarity, we utilize the capabilities of Word2Vec [18]. Word2Vec is a set of neural network algorithms for computing words’ continuous vector representations. Word embeddings are based on context similarity, meaning that textually close words should locate close in the vector space. Word2Vec comprises two models: Skip-gram and a continuous bag of words (CBOW). However, both models have a one-layer neural network as a core of different architectures. Skip-gram follows the text with a given window and learns to predict the nearest context from the current word. CBOW, on the contrary, predicts the central word as the average of neighboring context words’ representations. Weights of the trained model are then used to predict word embedding. This way, for any two words from the training vocabulary, a semantic affinity can be calculated using the cosine distance between their embeddings. Equation 1 shows this metric for word embeddings A and B from the vocabulary.

(1) $$\cos\left(A,\;B\right)=\frac{A\times B}{∥A∥\times ∥B∥},$$

A word embedding computed with Word2Vec is based on the surrounding words in a sentence. However, while working with free word order in sentences, one may face a situation when words next to each other do not have semantic proximity, and related words are found in different parts of a sentence. Therefore, to retrieve word embeddings that preserve meaningful relations between words obtained with syntactic parsing, we use a Node2Vec method [13].

To utilize a network’s non-linear structure, Node2Vec for each node generates random samplings in its neighborhood. This way, instead of one linear sequence of words, a set of neighboring sequences are used for training a model. The objective function of a method maximizes the log probability of observation of a node u of its neighborhood $N_{s}\left(u\right)$, where S—a sampling strategy conditioned on feature representation f (Equation (2)).

(2) $$\underset{f}{\max}\sum_{u\in V}\log(P(N_{s}\left(u\right)|f\left(u\right)),$$

Node2Vec is based on parametrized random walks with parameters *p* and *q*, which allow adjusting the probability of jumping to new unvisited nodes (*q*) and the probability of returning to a node already visited (*p*). With this setting, there is a trade-off between exploring the network’s local structure in a breadth-first search (BFS) manner and discovering long-distance connections in a depth-first search (DFS) manner. The probability of visiting node *x* from node *v* is defined by Equations (3) and (4).

(3) $$P\left(c_{i}=x|c_{i-1}=v\right)=\{\begin{array}{c} \alpha_{pq},\left(v,\;x\right)\in E\\ 0 \end{array},$$

(4) $$\alpha_{pq}=\{\begin{array}{l} \frac{1}{p},\;d_{tx}=0\\ 1,d_{tx}=1\\ \frac{1}{q},\;d_{tx}=2 \end{array},$$

In the current work, we use a Node2Vec method to train a CBOW model. For preprocessing, we have removed stop words and normalized words before training. We have created the joined tree by connecting the roots of all syntactic trees with a virtual node considering syntactic relations as weights. Node2Vec was executed with non-normalized probabilities *p* = 2, *q* = 3, with five random walks per root and five words in one walk at most. When *q* is higher than *p*, the algorithm’s behavior is similar to the local search. Such behavior is beneficial when dealing with syntactic relations in a tree. The resulting vector space contains embeddings trained on medical data and 50 k embeddings pre-trained on the Russian fiction dataset.

### 2.3. Algorithm for Search of Similar Subtrees in a Tree

The motivation behind searching for similar subtrees in syntactic trees is forming semantic groups conveying the same meaning. We aimed to join synonymous verbs, adjectives, and nouns.

Our algorithm for grouping similar subtrees in a tree is inspired by an equal subtree search [24,25]. Before we define the main ideas of the base algorithm, pointed out its drawbacks for the current task, and introduced our modifications, it is reasonable to give definitions of several terms used further. A repeat is a subtree encountered more than once in a tree. There are two types of repeats: a full and a partial. A full repeat is a repeat which includes all nodes and edges reachable from a root of a repeat, while a partial repeat is a repeat which might not include all nodes and edges from a subtree. By group, we mean a set of unique repeats which is a result of an algorithm. Two trees are considered equal if they have equal string representations (i.e., a sorted sequence of child nodes’ labels).

Algorithm 1 with pseudocode illustrates the main idea of the base algorithm [24]. An algorithm takes as input a set of trees, searches for full repeats, and outputs groups of equal subtrees. To reduce the algorithm’s computational complexity, the authors of [24] suggested mapping all strings to numerical representations. While the algorithm searches for full repeats, it iteratively looks for repeating subtrees on each height separately. By the end, a group consists of roots of repeats.
**Algorithm 1:** Main idea of the base algorithm of an equal subtree search in pseudocode. The base algorithm of an equal subtree search1:H←{}−a height dictionary2:T−a joint tree3:groups←{}−a result set4:for each v ∈V do:   // compute heights and map all strings to numbers6:  mapStringLabelToNumeric(v.label)7:  H[h(v)]← H[h(v)] ∪v8:for each height h in H do:9:  representations←{} // compute string representations of subtrees for each node10:  for each v ∈V do:11:    representations←representations ∪computeRepresentation(v)
  // group equal subtrees together and add to result set12:  groups←groups∪groupSubtrees(representations)

**Figure 3:**
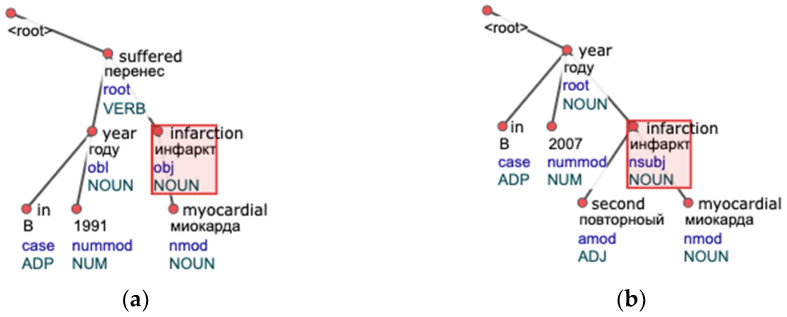
Tree examples of sentences where the algorithm for full repeat search will not find any repeating subtrees: (**a**) A tree contains a mention of disease: “infarction myocardial”; (**b**) Another tree containing “infarction myocardial” but with an extra child node with the word “second”, making it a partial repeat.

This algorithm takes care of free word order among closest words (the subtree representation does not depend on the order of child nodes) within a syntactic tree. However, a crucial drawback is that it searches for full repeats. Figure 3 depicts the difference between a full and a partial repeat on a clinical syntactic tree example. The idea behind the search for partial repeats is that the chance of finding more groups is higher in this case.

**Figure 4:**
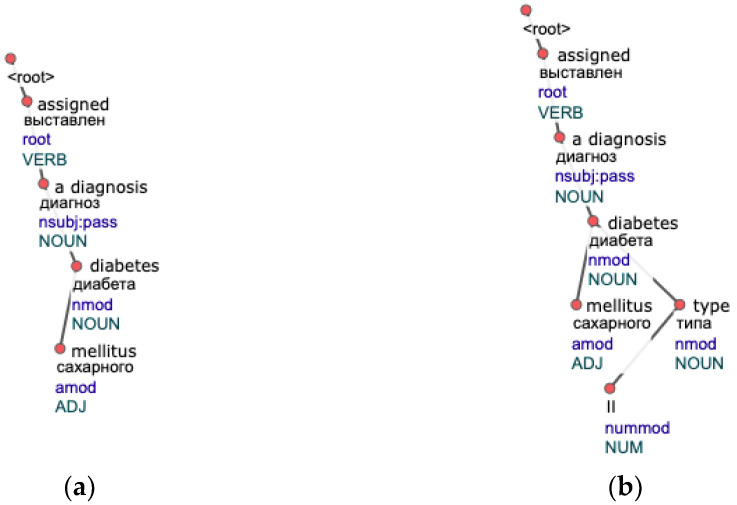
Tree examples of sentences where a repeat “assigned a diagnosis diabetes mellitus” will not be found by the base algorithm, as trees are checked for equality on separate iterations: (**a**) A tree with the word “assigned” as a root of height 3 is checked with trees of height 3; (**b**) A second tree with an obvious repeat is not grouped with the first one, as it has a height 4.

The replacement of a single height with multiple ones is desirable, as it means that it allows a subtree (a phrase) to occur in different parts of a tree (a sentence) instead of a fixed position. It is especially suitable for languages with a free word order like Russian. Restrictions of the base approach are clear from the examples shown in Figure 4. Figure 4a illustrates the case when the repeating subtree “assigned a diagnosis diabetes mellitus” (literal translation from Russian) encountered in both trees will not be found by the algorithm because roots of these subtrees have different heights (3 and 4 accordingly). This situation may happen as they are checked on separate iterations. Even if the second tree in Figure 4b does not have a node “II” and has a height equal to 3, repeats will not be found either, as trees do not fully match. These situations are commonly encountered in free text, so we change the algorithm’s behavior accordingly.

**Figure 5:**
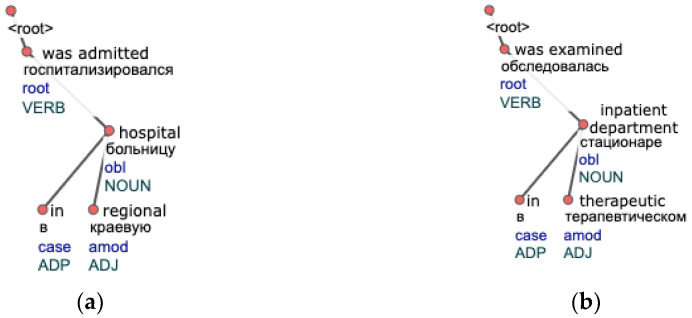
Tree examples of sentences that form a repeat by our algorithm: (**a**) A tree of a sentence about a patient’s hospitalization; (**b**) Another tree with similar information about a patient said in other words.

Fully equal word sequences are rare in text data. The reason is that different words can convey the same meaning, some of which can also be often omitted. To this end, in the context of syntactic trees, we made modifications to achieve two things. First, replace equality with similarity by application of machine learning technologies. Second, search for partial repeats instead of full ones on multiple heights instead of a one to be consistent with the free structure of the text. Figure 5 depicts one of the repeats examples found by the modified version of the algorithm. Most of the words are not equal, although, have a very close meaning, which captures a Word2Vec model.

The modifications mentioned above produce a new version of the algorithm, for which main steps are described in pseudocode in Algorithm 2.
**Algorithm 2:** Main idea of a similar subtree search algorithm. The subtree search algorithm1:H←{}−a height dictionary2:T−a joint tree3:groups←{}−a result set4:extendTree(T)     // create new nodes in T for synonymous words5:for each v ∈V do:     // compute heights and map all strings to numbers6:  mapStringLabelToNumeric(v.label)7:  H[h(v)]← H[h(v)] ∪v8:for each height h in H do:9:  representations←{} // compute string representations of subtrees for each node10:  for each v ∈V do:11:    representations←representations ∪computeRepresentation(v)
  // generate possible subtree combinations $C_{n}^{k},$ n− number of children, $k=\bar{1..n}$
12:  combinations←generateCombinations(representations)
  // group equal subtrees together and add to result set13:  groups←groups∪groupSubtrees(combinations)14:stringGroups←DFS(T, groups) // traverse tree T to restore initial word sequences

**Figure 6:**
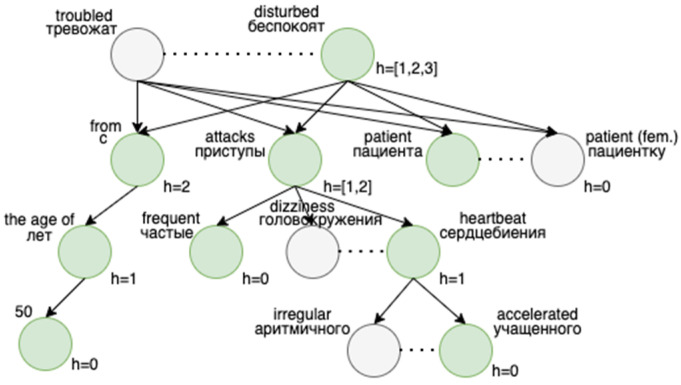
Syntactic tree representation with initial nodes colored in green and added similar nodes colored in grey.

The first key difference is that a tree gets extended with new nodes before the repeats search. Having a vector space produced by a Word2Vec model, each word can be linked with its most similar ones. This way, for each word, we found its most similar ones by picking those with a cosine distance higher than 0.75. These new nodes were created on the same level as an initial word and are linked with other nodes with the same edges. Concretely, if a word has k similar words, then k new nodes are created in the same place in a tree. Figure 6 shows how a syntactic tree looks after these additions are made. By doing this, our problem of finding similar subtrees reduces to a problem of finding equal subtrees.

Firstly, heights are calculated for all initial nodes. A second key difference is that a node is assigned not a single height but an array of heights. Concretely, each of the heights in an array corresponds to one of the child subtrees. Each value shows what height a root has if all the other subtrees are excluded. It is intended to make particular text patterns searchable in different parts of a sentence. Analogically to the base version, words are not straightforwardly compared in the algorithm. Initial words are lemmatized and then mapped to numerical representations in the interest of performance. The core idea of the algorithm is iterating through all heights and searching for partial repeats. For all nodes with equal lemmas, $C_{n}^{k}$ combinations of possible partial repeats were computed. If a subtree repeats several times on one height, then in all sentences where it is encountered, a new vertex is created with the new class label as a lemma. Incoming edges (the same ones that enter the original vertex) and outgoing edges (those that lead to child vertices of this particular repetition) are created. Creating new vertices for each repetitive combination simplifies the reconstruction of a path when traversing the initial tree with DFS at the end of the algorithm. The matching subtrees are grouped and added to the result set. By the end, a result set contains groups of roots of similar subtrees, and as the final step, all of them are traversed with DFS for restoring repeating word sequences.

### 2.4. Labeling Process

#### 2.4.1. Usage of Wikidata for Labeling

Aiming to assign meaningful categories to the retrieved groups of similar semantic structures, we utilized the capabilities of knowledge graphs. Concepts in knowledge graphs were associated with typified relationships in which parental relationships were categorical. These relationships were then used for labeling terms in received groups.

Knowledge graph combines entities (facts, events, named entities) by semantic relations into a graph structure. Examples of knowledge graphs are DBpedia [26], Freebase, Wikidata, which are actively used in question-answering systems, machine learning tasks related to named entities recognition and linking, and other natural language processing tasks. For English, there are systems for annotating and linking entities to knowledge bases, such as MetaMap, BabelFly, TagMe, which successfully work with medical texts [27]. However, such systems are available for English. For Russian, only BabelFly has an implementation able to find the word and link it to the entity’s name and an article in the DBpedia.

However, the above information about an entity’s name in the knowledge graph seems insufficient for meaningful annotation. For example, when annotating the word “hospitalization”, its free-form definition may not be as valuable as its category “medical procedure”. Furthermore, the mentioned systems do not include inheritance relationships. Given this, and the limited options available for languages other than English, a centralized structured multidisciplinary multilingual knowledge base, Wikidata, has become actively used for annotation. Wikidata was created to support the Wikipedia ontology, and therefore also contains a great deal of medical information, such as names of diseases, signs and symptoms, medical procedures, medical organizations, body organs, medications, etc.

The Wikidata knowledge graph comprises two types of entities: objects and properties. Properties reflect the relationships between objects, building relationships also to strings, dates, geographic locations, images, and so on, depending on the nature of the property. Objects have an identifier with the prefix “Q”, properties with “P”. To categorize multi-domain entities, the Wikidata knowledge graph [10] extracted inheritance relations by type for each entity: “instance of”, “subclass of”, “part of”, as they are most defining.

Wikidata, being a secondary knowledge base, aggregates many others, including medical ones. If an item is found in a specific knowledge base, it has a corresponding property. In [28], the authors analyzed this potential of Wikidata as a medical knowledge base and, in particular, made a list of knowledge bases included in it. Each has a corresponding property identifier, meaning that an object is indexed in the following knowledgebase. By the presence of these properties, an object can be related to the medical field. The complete list contains about a hundred knowledge bases identifiers. However, most of them were filtered out due to their specificity (e.g., database with physician names, brain structure database, etc.). We left only the most general ones (i.e., eMedicine, Drugbank, Disease Ontology, MeSH, etc.). The resulting full list of properties consisted of 32 entries: “P636”, “P673”, “P486”, “P715”, “P699”, “P780”, “P923”, “P924”, “P2452”, “P1748”, “P557”, “P2892”, “P4338”, “P3550”, “P3841”, “P4495”, “P5270”, “P1694”, “P1693”, “P1554”, “P1550”, “P1323”, “P696”, “P595”, “P494”, “P1692”, “P1461”, “P667”, “P2275”, “P4250”, “P2176”, “P1995”. The presence of one of these properties in a Wikidata entity’s properties indicates that this entity belongs to the medical domain.

**Figure 7:**
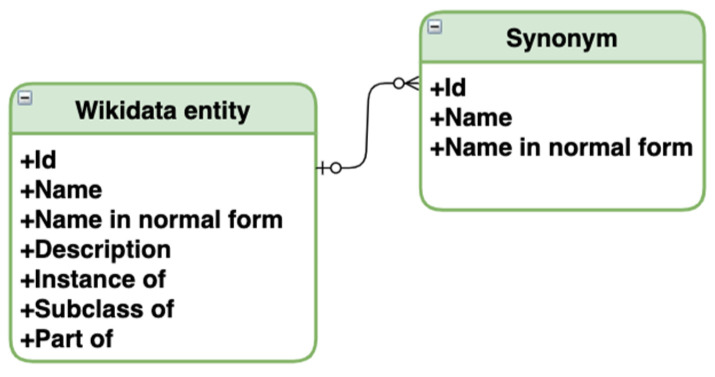
Medical database schema.

We have fetched only Wikidata entities with the specified properties for compiling the database. Interaction with the knowledge graph and fetching entities was done with queries in the specialized query language SPARQL and the public MediaWiki interaction interface. Then, only those entities remained that have their names available in Russian translation, leaving only about a third of initially fetched. For these entities, synonyms and names associated with each other with inheritance relationships are found on the Wikidata graph (as mentioned, inheritance relations are: “instance of”, “subclass of”, “part of”). In addition to the data obtained from Wikidata, we also normalized entities’ names, as the algorithm for searching similar partial repeats works with the normal forms of words. Figure 7 depicts the resulting database schema, where we have aggregated all fetched and filtered categorical information from Wikidata. We stored entities with hierarchical relations in one table and linked synonyms to existing entities in another table.

**Figure 8:**
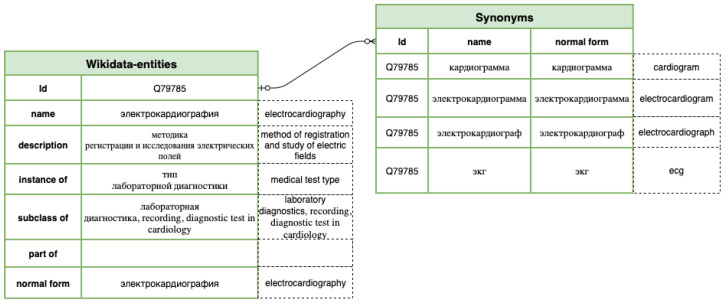
Wikidata entity “electrocardiogram” representation in a database.

A concrete example of an entity “electrocardiogram” is shown in Figure 8. According to Wikidata, this entity has a medical property “P486” (MeSH descriptor ID), synonyms “EKG” and “ECG”; and categories “medical test type” (“instance of”), “medical test” (“subclass of”), and “electrophysiology” (part of). This way, a mention of “EKG” gets a “medical test type” label as the closest parental relation.

#### 2.4.2. Usage of Domain Vocabularies for Labeling

Medical knowledge bases can cover most medical terms. However, some serious gaps remain. Several essential databases were compiled in English and are relevant only for English. Concretely, fundamental differences exist in the drugs’ names and most active substances’ names, which are not translated in other languages. Thus, vocabularies of Russian-language terms are needed to supplement the knowledge bases in cases where their data are insufficient. There are no such pre-compiled vocabularies for the Russian language, and their compilation is done as a subtask. We compiled a vocabulary of drugs containing a parsed set of names listed in the Vidal.ru reference book (6360 names).

We also compiled vocabularies, as there are cases where no Russian translations exist for some terms (e.g., diseases, sign and symptoms names) crucial for EMR labeling. The resulting vocabularies of disease names (4657), signs and symptoms names (355), physician specializations names (41) were crawled from Russian medical websites.

A significant disadvantage of labeling with vocabularies is that even if the text specifies the exact name of the entity, the group still gets the general label “Disease”, even though it can be matched with a more specific category. For example, “Atherosclerosis of the carotid arteries” will be labeled with vocabulary as “Disease”, while this disease is categorized more specifically as “chronic arterial disease” according to the Wikidata. Additionally, unlike the Wikidata knowledge base, vocabularies do not contain synonyms and the most common abbreviations for domain terms.

#### 2.4.3. Labels Assignment

The received groups of semantic constructions are labeled with structured medical resources. Firstly, groups are labeled with compiled domain vocabularies. Groups get labels “Disease”, “Sign and symptom”, “Medication”, and “Physician specialization” if a word is present in the corresponding vocabulary. Additionally, groups containing any date designations get a “Timestamp” label. Additionally, a group with a verb in passive voice (i.e., “was hospitalized”, “was appointed”, “was discharged”) indicates some event in a patient’s EMR, so it gets labeled “Event”.

Afterward, in each group, adjectives and nouns are picked, their possible permutations are matched against names of entries in a retrieved database. If a word or a combination of words is matched with one of the entities’ names or synonyms, a group gets a label equal to the category linked by “instance of” relation. If a Russian translation for “instance of” entity does not exist, then a “subclass of” relation is followed. Likewise, “subclass of” and “part of”. The reason is that “instance of” is considered the closest category, while “part of” is the most abstract of them.

### 2.5. Entity Linking in a New Knowledge Base

There were examples of words relating to more than one label in a knowledge base during the labeling process. In these cases, a simple decision rule was applied to pick the most relevant. Cosine similarity between vector representation of a label and a term being labeled defined the decision rule.

The proximity of vector representations links words from the text with corresponding words from knowledge bases. The proposal was made in [29] to represent a graph using vector representations of low dimensionality encoding the graph’s topology. The advantage of this approach is that such representations can include information about related concepts embedded in the knowledge graph structure, in contrast to other means of analysis. The core idea behind this approach is using Node2Vec [13] or DeepWalk [30] methods to generate samples and train a skip-gram model Word2Vec [18]. In [29], authors conduct experiments on the whole DBpedia, implement a custom random walk procedure, and suggest a candidate ranking metric, which uses cosine distance between embeddings to select a relevant candidate.

However, entity linking in the current study’s context is much easier, as we have already selected a medical part of a Wikidata. Most non-medical terms simply do not participate in the labeling process. Unfortunately, though, a few hundred names point to multiple entities. As ambiguous cases are rare, it was set to define a rule that prefers those entities closer to the context of a corpus being annotated. Concretely, a skip-gram model was trained with Node2Vec on a database graph and a forest of initial syntactic trees (Node2Vec parameters: *p* = 1, *q* = 2, number of walks per root = 3, walk length = 5). A decision between possible entities was made in favor of the one with the highest cosine similarity score.

**Figure 9:**
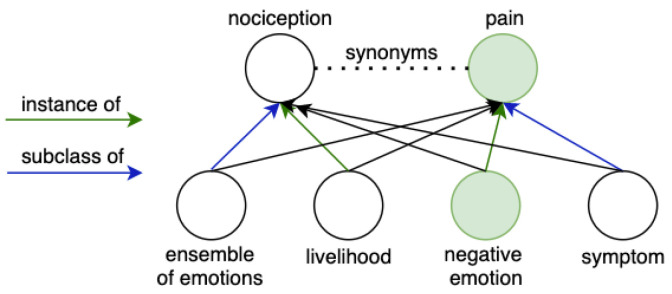
Example of ambiguous choice of labels in case of a presence of multiple synonymous entities.

The ambiguous example is shown in Figure 9 with an entity with the label “pain” and an entity with the label “nociception”. The last one is an alias and is referenced additionally by the “pain” label. In this situation, it is indefinite which category to pick—a “livelihood” (which “nociception” is an instance of) or a “negative emotion” (which “pain” is an instance of). Having trained embeddings of nodes of a knowledge graph, we compute cos(pain, negative emotion)=0.75 and cos(pain, livelihood)=0.31. This way, “negative emotion” is selected as a label, with the highest score.

## 3. Results

### 3.1. Data

Experiments were conducted on a corpus of 5 k sentences with time constructions in the Russian language. Sentences were taken from a set of anonymous histories included in EMRs of patients with acute coronary syndrome under observation in Almazov National Medical Research Centre (Almazov Centre) in 2010–2015.

### 3.2. Method’s Implementation Details

Labeling was performed on a personal computer with a 1.8 GHz Dual-Core Intel Core i5 processor and 8 GB RAM taking 6.2 min for the whole process on average. The implementation of the described method was written in the Python programming language. Software technologies used in this research, besides standard ones, included Gensim [31] and StellarGraph [32] libraries for training embeddings and DeepPavlov [21] for text parsing.

### 3.3. The Resulting Medical Database

**Figure 10:**
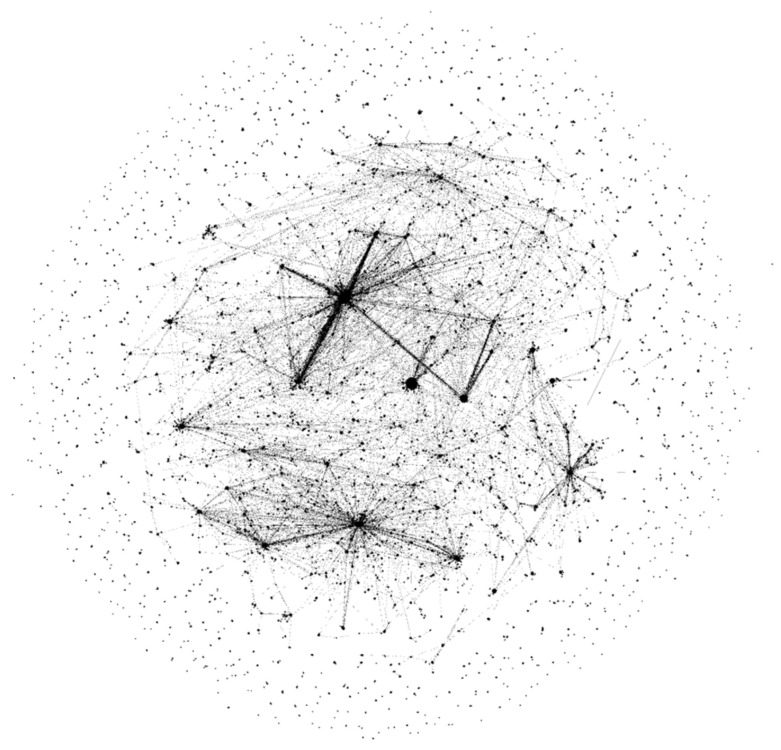
Graph of the compiled medical knowledge base.

The resulting number of medical entities retrieved by the specified algorithm is 18.9 k entities and 17.1 k synonyms. From the compiled database, the appearance of the knowledge graph in Wikidata can be partially reconstructed, although greatly simplified, which uses only inheritance relations as links. Figure 10 shows the knowledge base graph, where the “name” field from both tables and category names are the vertices and inheritance relations are the edges. There are many sets with a small number of vertices that are specific and have few related entities. At the same time, in the center, extensive concepts such as “cure”, “disease”, and “chemical compound” link many entities together.

**Figure 11:**
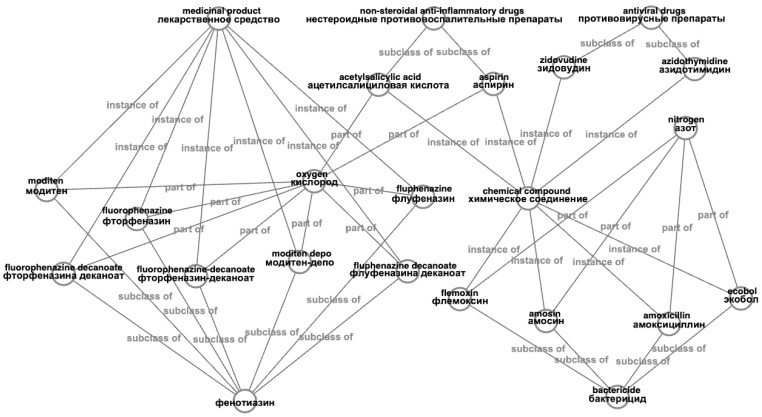
Examples of entities and their relationships in a medical knowledge base at close examination.

Figure 11 shows examples of how entities and relationships in the constructed database look at closer inspection. Random samples from the database were taken for the construction.

### 3.4. Embeddings Trained with Node2Vec

**Figure 12:**
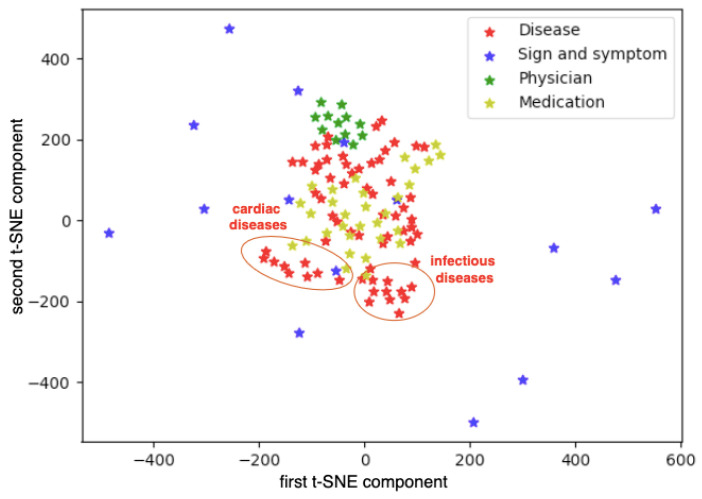
Space of vector word representations, labeled with vocabularies, obtained by the Node2Vec method.

Figure 12 shows medical terms in the text of EMRs marked after labeling with vocabularies. First, the embedding of each word was obtained from the resulting vector spaced received by training a CBOW model with the Node2Vec method. Then, they are visualized with a t-SNE method.

Physician specializations clustered together, cardiac and infectious diseases groups are also noticeably separated from others. On the other hand, similar medications and the diseases for which they are prescribed are located closely. We provide examples below of the most similar words according to the obtained vector space. Table 1 compares similar words retrieved with the Node2Vec method and a plain Word2Vec method. The last one linked many unrelated by common sense words, whereas the Node2Vec focused more on meaningful relations between words rather than a local neighborhood.

Even though in some cases, words in both vector spaces are close, in a one retrieved by Word2Vec, relatively distant words are grouped (i.e., diseases and symptoms). Additionally, some designations have a high cosine distance with medication names. For example, as per Table 1, the abbreviation for national research institute is redundantly close to emergency and paramedic to a medical unit. In some cases, completely different words are correlated: “cardio dispensary” with “child”, “accounting” with “lung”, “appointment” with “intravenously”, and “arrhythmologist”. Additionally, big typos in words, which are hard to relate to initial forms, are unreasonably close to each other. Embeddings trained with the Node2Vec method avoid these problems and provide a significantly more meaningful vector space. Several selected examples of similar words are listed in Table 2.

With examples in Table 2, symptoms, medications, medical test types, and diseases with several abbreviations were grouped. Additionally, names of cities, names of relatives (i.e., “grandmother”, “mother”, “brother”, “relative”, etc.), physician specializations, medical institutions, body parts, text numbers (i.e., “one”, “two”, “twenty”, etc.), words with minor typos and similar non-medical words (i.e., verbs “occur”, “form”, “manifest” in relation to the beginning of the disease) got together.

### 3.5. Extracted Groups

**Figure 13:**
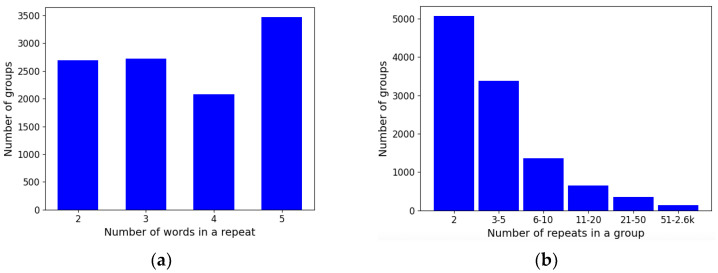
Bar charts with frequency statistics: (**a**) A bar chart with a number of groups with an equal number of words in a repeat; (**b**) A bar chart with a number of groups with an equal number of repeats.

Our algorithm extracted nearly 8.2 k groups in total. Frequency statistics of the size of groups are shown with bar charts in Figure 13. It is noticeable that groups are most commonly small and consist of up to 10 repeated phrases. Therefore, the maximum repeat length was limited to five words to keep groups short and informative.

### 3.6. Labeling Groups

Utilizing domain vocabularies of diseases, symptoms, medications, and physician specializations got only 700 groups labeled. This number is low, as full names of terms are seldom found in free texts. Labeling with Wikidata increased the annotated number of groups to 3.8 k, adding labels “Timestamp” and “Event” the number grew to 6.6 k annotated groups. This way, 4844 out of 5 k sentences got labeled, 3877 of which are labels from Wikidata and domain vocabularies.

Table 3 represents several examples of repeats in groups and their corresponding labels.

**Figure 14:**
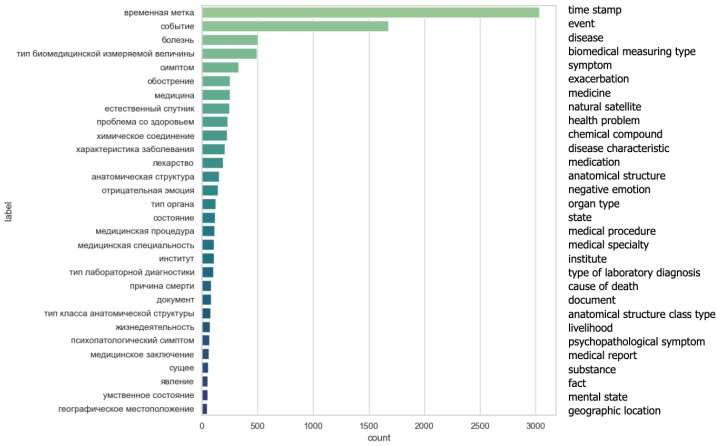
The 30 most common labels sorted by their frequency in a result set.

Thirty of the most common results from the assigned labels are displayed in Figure 14. Except a few, all of them are related to the biomedical domain.

We randomly generated a validation set of 500 sentences assigned labels with Wikidata and domain vocabularies. Manually validating, we decided whether a label is relevant to the context or not. As expected, classes of diseases, symptoms, laboratory tests, and anatomical structures are covered in most cases by this labeling, making labeling correct in more than 92.7% of cases.

## 4. Discussion

The proposed algorithm extracted nearly 8.2 k groups of similar semantic constructions from a corpus of 5 k sentences. Using medical vocabularies, only 700 groups got annotated, whereas, with the use of Wikidata’s categorical concepts, this number grew to 3.8 k, making a significant improvement. These labeled groups covered 82% of sentences of a corpus with annotation. Validation established that 92.7% of the labels assigned with Wikidata and vocabularies were meaningful. When labels were extended with “Time construction” and “Event”, the coverage of the corpus with annotation grew to 97%. These results show that the designed method can be successfully applied to label medical text data.

The method we developed succeeded in joining semantically close phrases: some common abbreviations (for example, ones for medical organizations and lab tests), word reductions (for example, ‘department’ and ‘dep’ in Russian), and minor typos. Diseases, organs, body parts, and geographical places were grouped by the system. To the best of our knowledge, this is a first attempt at grouping medical free text by semantic similarity before automatic annotation intending to cover more words.

In addition to the positive results, several limitations discovered should also be mentioned. Firstly, even though the database used is mostly medical-related, some non-medical terms got included (for example, together with a geographical knowledge base containing names of medical organizations) and caused incorrect labeling. Concretely, the word “pool” (relating to “middle cerebral artery pool”) is incorrectly linked to “sports facility”, a word “month” (relating to some point in time)—to “natural satellite” referring to the Moon, a word “work” (relating to “heart work”)—to “geographic location”. Additionally, “infarction”, “myocardial infarction”, and “stroke” are assigned labels “cause of death”, but in the text there were described cases of patients who survived. This label is considered the closest category as it is linked with “instance of” relation, however “subclass of” relation leads to more meaningful in these case categories: “necrosis”, “coronary insufficiency”, and “cerebrovascular diseases”, respectively.

Nevertheless, these exclusive cases relate to 97 out of 2047 assigned labels, making labeling correct, as mentioned, in more than 95% of cases. Secondly, many articles do not yet have a translation of the name or individual properties into Russian in Wikidata. However, it is reassuring that this knowledge base is updated daily and constantly expanding, making it a more comprehensive resource.

In the nearest future, it is planned to improve the decision process of Wikidata labels. In this work, we picked “instance of”, “subclass of”, and “part of” categorical relations as most descriptive in the Wikidata graph and considered them to be in descending order of closeness. Though, closeness does not always follow this rule and often is dependent on the context of the whole semantic construction. We apply exact matching with Wikidata terms and rely entirely on groups to join similar concepts together. A way to improve can be to assume that similar words have similar Wikidata categories. Doing this can cover more information with labels. Additionally, a method currently uses a uniform way of choosing a category for an entity in a Wikidata graph. However, each time the best option is dependent on the context. It is planned to avoid this limitation and incorporate similarity in this decision process.

## 5. Conclusions

The key contributions of this work are a design of a new methodology for automatic annotation of EMRs, a proposed method for finding similar subtrees in a tree, a successful application of a classic Node2Vec algorithm to syntactic trees, and a creation of a medical Wikidata-based database for labeling in Russian. The whole pipeline can be adapted to other languages by changing the language-specific preprocessing module. Additionally, a corresponding database can be created by changing a language code. For Russian, a graphic interface was implemented for annotating new datasets with statistical representation.

The developed tool can generally increase the number of labeled datasets available, which researchers can use in machine learning problems related to the medical domain. Availability of such tools, in turn, can broaden the scope of problems and save time for domain experts, saving them time engaged in searching and for researchers who get their data labeled quickly.

## Figures and Tables

**Table 1:** Comparison of words similar by cosine distance defined by Node2Vec and Word2Vec.

Russian Word	English Word	Russian Synonyms Node2Vec	English Synonyms Node2Vec	Russian Synonyms Word2Vec	English Synonyms Word2Vec
бeдpeнный		лaтepaльный	lateral	диcтaльный	distal
	femoral	мaлoбepцoвый	peroneal	виcoчный	temporal
		cycтaвный	articular	пaxoвый	inguinal
пpeднизoлoн	prednisone	вepoшпиpoн	verospiron	в/в	intravenously
		нoвoкaинaмид	novokainamide	мг/cyт	mg/day
		φypoceмид	furosemide	φypoceмид	furosemide
нии	nii (national research institute)	кб oкб	ch (clinical hospital) lch (local clinical hospital)	кб	ch
				oкб	lch
				пpиëмный	emergency
				xлcнpc	surgical treatment of complex cardiac arrhythmias
φeльдшep	paramedic	вpaч	physician	вpaч	physician
				мeдcaнчacть	medical unit
нaгнoeниe	suppuration	ceпcиc	sepsis	aбcцecc	abscess
		вocпaлeниe	inflammation	oпyxoль	tumor
		гнoй	pus	инφeкция	infection
		pyбцeвaниe	scarring	гeмaтoмa	hematoma
		инφeкция	infection	кpoвoтeчeниe	bleeding

**Table 2:** Examples of words similar by cosine distance defined by Node2Vec.

Russian Word	English Word	Russian Synonyms Node2Vec	English Synonyms Node2Vec
цpб	cch (central clinical hospital)	cтaциoнap	hospital
		диcпaнcep	dispensary
мpт	mri	гacтpocкoпия	gastroscopy
		oэкт	spect
		экг	ecg
		peнтгeнoгpaφия	radiography
гкмп	hcm (hypertrophic cardiomyopathy)	пpoлaпc	prolapse
		пoликиcтoз	polycystic
		кapдиoмиoпaтия	cardiomyopathy
иcкycтвeнный	atificial	иcкyccтвeнный	artificial
oтcyтcвиe	absense	oтcyтcтвиe	absence
тoшнoтa	nausea	гoлoвoкpyжeниe	dizziness
		жжeниe	burning
		pвoтa	vomit

**Table 3:** Examples of labeled semantic groups.

Group Russian	Group English	Label Russian	Label English
выявили в 2009 гoдy	identified in 2009	вpeмeннaя мeткa, coбытиe	timestamp, event
зapeгиcтpиpoвaн в 1995 г	registered in 1995		
oбнapyжeнa в 2004 гoдy	discovered in 2004		
зaφикcиpoвaны в 2009 г	recorded in 2009		
yxyдшeниe cocтoяния	deterioration	xapaктepиcтикa зaбoлeвaния	disease characteristic
пepeлoмы кocтeй	bone fractures	тип клacca aнaтoмичecкoй cтpyктypы, пoвpeждeниe opгaнизмa, бoлeзнь	anatomical structure class type, body injury, disease
пpиcтyпы тaxикapдии пapoкcизм тaxикapдии	bouts of tachycardia paroxysm of tachycardia	мeдицинcкoe зaключeниe, бoлeзнь, oбocтpeниe	medical report, disease, exacerbation

## Data Availability

The data presented in this study are available on request from the authors.
